# The Cellular Protein MCM3AP Is Required for Inhibition of Cellular DNA Synthesis by the IE86 Protein of Human Cytomegalovirus

**DOI:** 10.1371/journal.pone.0045686

**Published:** 2012-10-19

**Authors:** Emma Poole, Mark Bain, Linda Teague, Yoshinori Takei, Ron Laskey, John Sinclair

**Affiliations:** 1 Department of Medicine, Addenbrooke's Hospital, University of Cambridge, Cambridge, United Kingdom; 2 Hutchison/MRC Research Centre, University of Cambridge, Cambridge, United Kingdom; 3 Department of Zoology, University of Cambridge, Cambridge, United Kingdom; Lisbon University, Portugal

## Abstract

Like all DNA viruses, human cytomegalovirus (HCMV) infection is known to result in profound effects on host cell cycle. Infection of fibroblasts with HCMV is known to induce an advance in cell cycle through the G_0_-G_1_ phase and then a subsequent arrest of cell cycle in early S-phase, presumably resulting in a cellular environment optimum for high levels of viral DNA replication whilst precluding replication of cellular DNA. Although the exact mechanisms used to arrest cell cycle by HCMV are unclear, they likely involve a number of viral gene products and evidence points to the ability of the virus to prevent licensing of cellular DNA synthesis. One viral protein known to profoundly alter cell cycle is the viral immediate early 86 (IE86) protein - an established function of which is to initially drive cells into early S phase but then inhibit cellular DNA synthesis. Here we show that, although IE86 interacts with the cellular licensing factor Cdt1, it does not inhibit licensing of cellular origins. Instead, IE86-mediated inhibition of cellular DNA synthesis requires mini-chromosome-maintenance 3 (MCM3) associated protein (MCM3AP), which can cause subsequent inhibition of initiation of cellular DNA synthesis in a licensing-independent manner.

## Introduction

As with all herpesviruses, human cytomegalovirus (HCMV) establishes lifelong persistence following infection. Although primary infection of healthy individuals is usually asymptomatic, infection or reactivation causes severe disease in immunocompromised individuals.

HCMV establishes latency in cells of the myeloid lineage and, following sporadic differentiation-dependent reactivation, disseminated lytic infection of multiple fully permissive cell types such as fibroblasts and endothelial cells occurs. Lytic gene expression follows a regulated cascade through three distinct phases – immediate-early (IE), early (E) and late (L) resulting in the release of infectious virions. The most abundant IE genes are IE72 and IE86 which arise from differential splicing of the primary major IE transcript. The viral IE genes have been ascribed many roles including autoregulation of the major IE promoter (MIEP) as well as regulation of cellular gene expression and many of the cellular genes that are upregulated by IE72 and IE86 are likely to optimize the cellular environment for viral replication [1]–[8].

Many studies have shown that HCMV infection perturbs normal cell cycle progression [9] and IE86 has been implicated in this process [10], [4], [11], [6], [12], [13]. In general, HCMV infection advances cells in G_0_-G_1_ through the G_1_-S phase checkpoint but then subsequently arrests cells in early S-phase.

This strategy, during the earliest stages of virus infection, is likely to ensure that any increases in pools of dNTPs etc, which would accompany viral-induced progression into early S phase, are then used efficiently for viral and not cellular DNA synthesis.

Viral functions associated with cell cycle advance, and in many cases their mechanism of action, have been defined. For instance, over-expression of HCMV-encoded pp71, IE72, IE86 or pUL97 in isolation inactivates pRb-family proteins and activates expression of E2F-dependent S-phase genes, thereby promoting G1/S- transition [14]–[20]. In contrast, the mechanisms by which HCMV prevents cellular DNA synthesis are less clear, although it has been shown that virus infection results in the inhibition of loading of MCM complex onto chromatin [21], [13]. MCM, is a hetero-hexameric protein complex composed of MCM2-MCM7 and is recruited to replication origins by Cdc6 and Cdt1 to form pre-replication complexes (pre-RC) in late M- to G1- phase of cell cycle resulting in replication licensing. Once activated by phosphorylation early in S phase, MCM functions as a replicative helicase to unwind the origin and initiate DNA replication [22]. Indeed, elegant studies have recently shown that viral pUL117 is, at least in part, responsible for this HCMV-mediated inhibition of MCM complex loading onto cellular origins thereby inhibiting cellular replication licensing [23].

However, little is known about the mechanisms by which other viral factors, such as pUL69 [24] and IE86 [12], [25], inhibit cellular DNA synthesis.

Some of the most profound effects on cell cycle during HCMV infection are mediated by the viral IE86 protein. Besides interacting with Rb family members, IE86 is also known to interact with other cell cycle regulatory proteins such as p21 and p53, which all helps to mediate advance of cell cycle through the G_0_/G_1_ phase check point resulting in an untimely entry into S phase [6], [26], [11], [4]. On the other hand, IE86-mediated inhibition of cellular DNA synthesis and concomitant cell cycle arrest are profound and have been shown in the context of both virus infection [12] and in isolation[4], [13], [27]. However, there are conflicting accounts of the role IE86 on cell cycle arrest during HCMV infection [28] and the mechanism by which IE86 does this is unclear.

Here we show that, although IE86 physically interacts with Cdt1, which is a critical component for the licensing of DNA synthesis at cellular origins of replication, this interaction does not interfere with the ability of Cdt1 to orchestrate licensing of cellular replication origins. Instead we show that IE86-mediated inhibition of cellular DNA synthesis requires MCM3AP, a cellular factor which has been shown to inhibit initiation, but not licensing, of cellular DNA synthesis after its recruitment to the nucleus by MCM3 [29], [30].

## Results

### Viral IE86 interacts with the cellular pre-RC licensing component Cdt1

**Figure 1:**
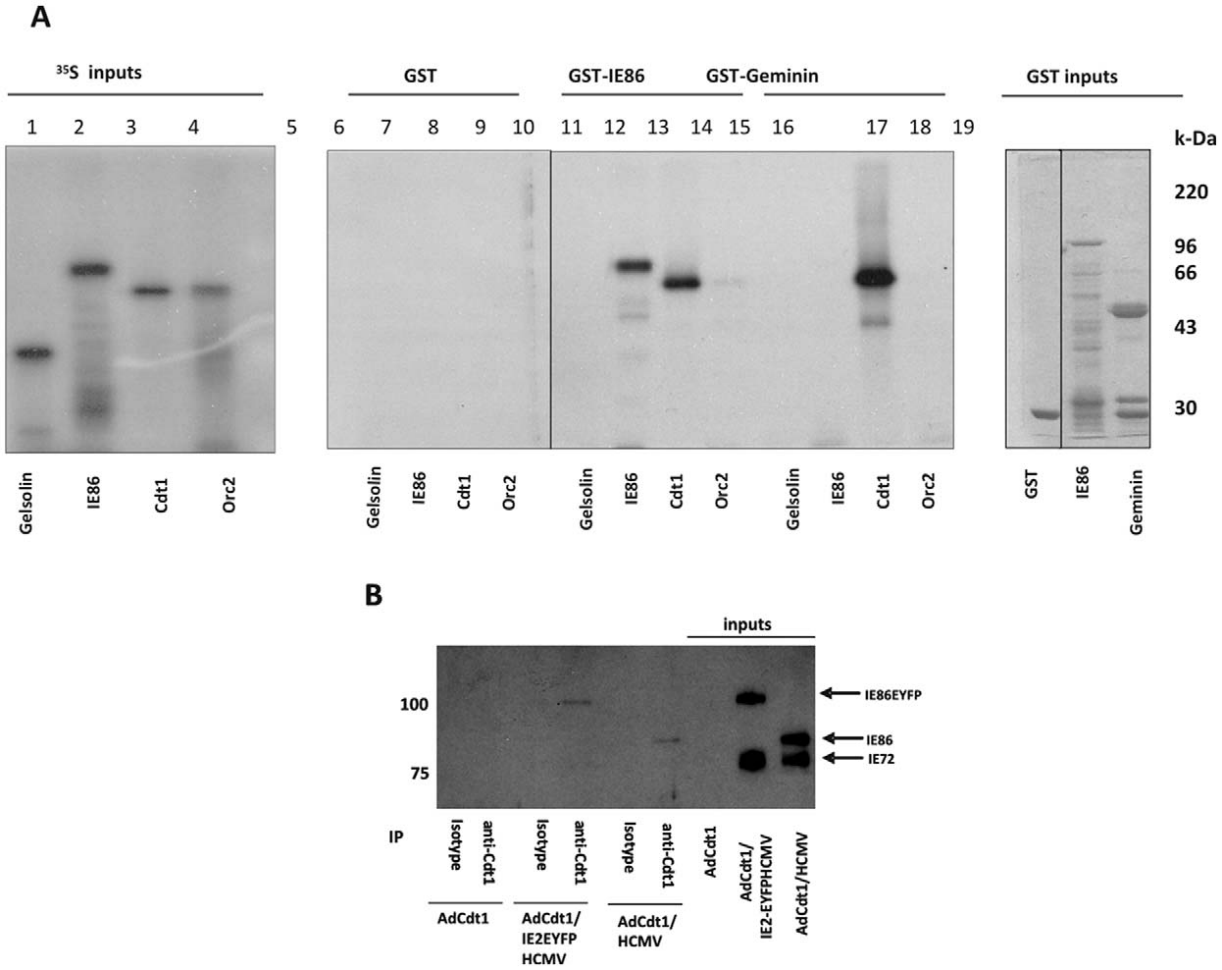
Viral IE86 protein interacts with pre-RC component Cdt1. (A) ^35^S-methionine -labelled cellular pre-RC components Cdt1 and Orc2 as well as IE86 and gelsolin were expressed in rabbit reticulocyte lysates (inputs lanes 1–4) and tested for their ability to bind to GST (lanes 5–8), GST-IE86 (lanes 9–12) or GST-Geminin (lanes 13–16) as indicated. 1/10 input proteins are also shown (lanes 1–4) as well as GST inputs (lanes 17–19). (B) Alternatively, HFFF cells were co-infected with 1 pfu/cell AdCdt1 and 5 pfu/cell of HCMV or HCMV expressing IE86-EYFP for 48 h followed by immunoprecipitation with anti-Cdt1 antibody or a matched rabbit serum control and then western blotted with E13 (which detects both IE72 and IE86). Input proteins are also shown which clearly distinguish between IE72, IE86 and IE86-EYFP.

The formation of a replication complex (RC) on cellular origins of DNA replication which is competent for initiation and elongation of DNA synthesis is a highly ordered affair [31] and has been termed licensing. The requirement for a step-by-step assembly of licensing factors to generate a pre-replication complex (pre-RC) provides numerous points at which HCMV-IE86 could inhibit cellular DNA synthesis. One of the first of these is the recruitment of Cdt1 - an essential early step that follows Orc binding in the ordered recruitment of cellular factors which assemble the pre-RC to license cellular replication origins. To test whether IE86 could be involved in the inhibition of cellular DNA synthesis at the very earliest times of pre-RC formation, perhaps by interaction with components of the pre-RC itself, we carried out GST-fusion pull down assays using IE86 as bait and various ^35^S-labelled components of the pre-RC (Figure 1A). As expected [32], GST-Geminin was able to bind Cdt1 but not Orc2 (lanes 15 and 16 respectively) or an irrelevant control protein such as gelsolin (lane 13). Interestingly, although IE86 did not bind Geminin (lane 14) or Orc2 (lane 12), it was able to complex with Cdt1 (lane 11). As expected, IE86 was also able to interact with itself (lane 10) but not the negative gelsolin control (lane 9). This ability of IE86 to interact with Cdt1 in vitro was confirmed in vivo by immunoprecipitation assays of lysates from infected fibroblasts. Figure 1B shows that immunoprecipitation of Cdt1 from HCMV infected cells or, in addition, cells which were infected with an IE86-EYFP recombinant HCMV (to clearly distinguish between IE86 and IE72) resulted in clear co-precipitation of IE86 or IE86-EYFP, respectively. In addition, probing of the samples by western blot with an IE72-specific antibody confirmed the co-immunoprecipitated IE proteins to be IE86 (data not shown).

### The interaction between viral IE86 and cellular Cdt1 does not prevent Cdt1 or MCM3 from loading onto chromatin

**Figure 2:**
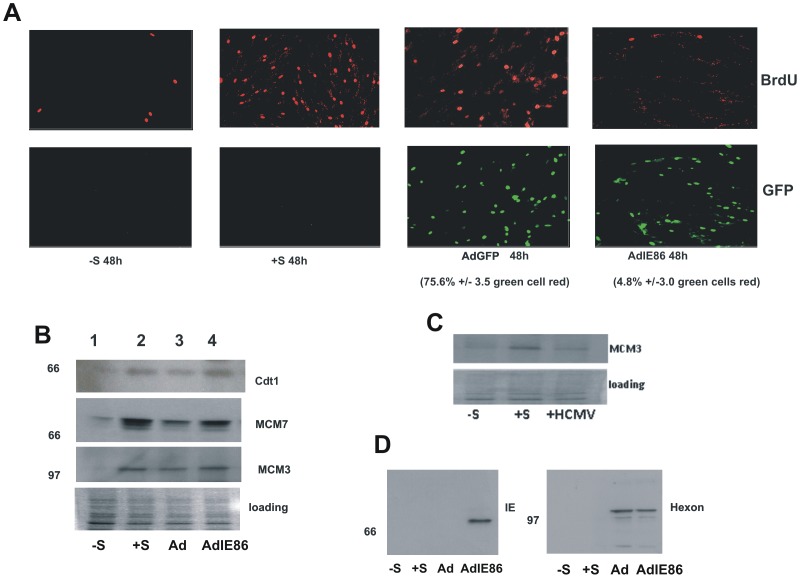
Inhibition of DNA synthesis by IE86 does not correlate with an inability to load MCM3 or MCM7. (A) Serum starved HFFF cells (−S) were treated with serum (+S) or treated with serum during infection with adenoviruses expressing GFP or IE86 (5 pfu/cell) as indicated for 48 h. After this time cells were co-stained for BrdU and IE protein or GFP as indicated. Quantitation of GFP or IE86 expressing cells which co-stained for BrdU incorporation using DAPI counterstaining (not shown) are indicated as averages +/−standard deviation from the mean from 5 independent fields of view. (B) Alternatively, serum starved HFFF cells (−S, lane 1) were treated with serum (+S, lane 2) or treated with serum during infection with AdGFP (Ad, lane 3) or AdIE86 (lane 4) as indicated and assessed for chromatin loading of Cdt1, MCM7 and MCM3. (C) MCM3 loading onto chromatin was assessed again in serum starved cells (−S) or serum stimulated cells (+S) in the presence of HCMV infection (HCMV). (D) Levels of IE86 expression and adenovirus hexon protein delivery in transduced cells were also analysed by western blot analysis.

As Cdt1 has been shown to be required for the subsequent recruitment of MCMs to cellular origins of replication to establish the pre-RC, we reasoned that an interaction between IE86 and Cdt1 could compromise MCM recruitment which would be consistent with the known ability of HCMV to prevent MCM loading onto chromatin of infected cells [21], [13]. To test this directly, we expressed IE86 in isolation in HFFF cells. We firstly confirmed that expression of IE86 did result in inhibition of cellular DNA synthesis as has previously been shown [6], [4]. We used previously published adenoviral expression vectors [6] to deliver IE86 to fibroblasts and analysed cellular DNA synthesis by BrdU incorporation (Figure 2A). As expected, serum-starved fibroblasts showed little evidence of DNA synthesis but stimulation of these cells with serum resulted in substantial staining with BrdU confirming induction of DNA synthesis. In contrast, and consistent with previous data [6], [4], IE86 expression but not control GFP expression resulted in profound inhibition of cellular DNA synthesis: 75.6%+/−3.5 of GFP-expressing cells continued to synthesise DNA whereas only 4.8%+/−3.0 of IE86-expressing cells stained positive for BrdU (Figure 2A).

On the basis that IE86 clearly inhibited cellular DNA synthesis, as expected [6], [4], we tested these cells for the presence of pre-RC factors on cellular chromatin as previously described [33] (Figure 2B). In these experiments, we ensured that good levels of IE86 were expressed specifically in ADIE86 transduced cells and that equivalent levels of adenovirus were used for control ADGFP and ADIE86 infections on the basis of delivery of adenovirus hexon protein to target cells (Figure 2D). As expected, serum-starved cells showed little evidence of Cdt1, MCM3 or MCM7 loading onto cellular chromatin consistent with their arrest in cellular DNA synthesis. In contrast, serum-stimulation of these cells resulted in new loading of Cdt1, MCM3 and MCM7, as expected (Figure 2B compare lanes 1 and 2, −/+ S). However, although IE86-expressing cells clearly showed an inhibition of cellular DNA synthesis (Figure 2A), Cdt1, MCM3 and MCM7 were still clearly recruited onto cellular chromatin (Figure 2B). In contrast, although IE86 in isolation did not result in a decrease in MCM loading (Figure 2B), HCMV infection did result in a decrease in loading of MCM3 (Figure 2C), as expected [13], [21], [23]. Therefore, although IE86 in isolation prevents cellular DNA synthesis, the mechanism by which this occurs does not appear to be by preventing pre-RC assembly.

### Viral IE86 interacts with the cellular protein MCM3AP

It has been shown that initiation of cellular DNA synthesis can be inhibited after assembly of the pre-RC by the MCM3 acetylase MCM3AP [34]. IE86 is known to bind to other known cellular acetylases such as PCAF as well as CBP and p300 [8]. Therefore, we asked whether HCMV IE86 was able to physically and functionally interact with MCM3AP and, if so, whether this interaction was involved in the mechanism by which IE86 inhibits cellular DNA synthesis.

**Figure 3:**
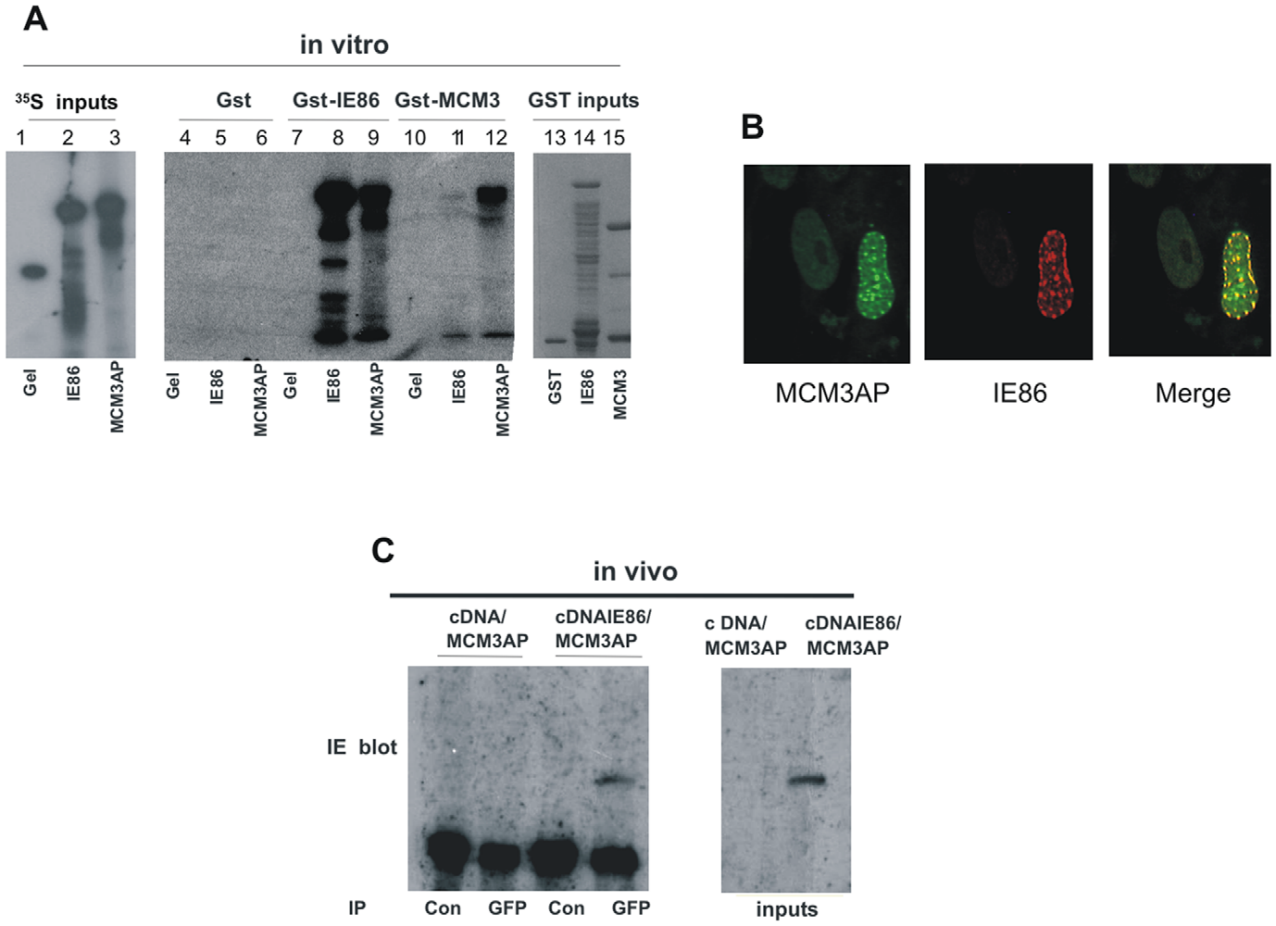
IE86 binds to MCM3AP in vitro and in virus infected cells. (A) ^35^S-methionine-labelled IE86, MCM3AP or gelsolin (Gel) were expressed in rabbit reticulocyte lysate (inputs shown in lanes 1–3) and tested for the ability to bind to GST, GST-IE86 or GST-MCM3 (inputs shown in lanes 13–15, binding shown in lanes 4–12). (B) Also, cells transfected with GFP-tagged MCM3AP (MCM3AP-GFP) were super-infected with HCMV and then analysed by immunofluorescence for IE86 using an IE86-specific antibody, SMX [52] and GFP-tagged MCM3AP. (C) To analyse interaction between MCM3AP and IE86 in vivo, HFFF cells were transfected with MCM3AP-GFP together with a cDNA3-GFP expression vector (cDNA) or an IE86 expression vector (cDNAIE86). Samples were immunoprecipitated with an anti-GFP (GFP) antibody or matched control antibody (Con) and co-complexed IE86 was detected by Western blot with anti-IE antibody (E13).

As a positive control, we first tested the ability of MCM3 to interact with MCM3AP. Figure 3A shows that using GST-fusion pull down assays and ^35^S-labelled proteins, consistent with previous findings [30], MCM3 is able to bind to MCM3AP (lane 12). We then tested whether IE86 also interacted with MCM3AP. Figure 3A clearly shows that, whilst IE86 appeared to bind weakly to MCM3 (lane 11), consistent with previous observations [25], a strong interaction was observed between IE86 and MCM3AP in vitro (lane 9). As before, we observed the relevant presence or absence of binding of IE86 to positive and negative controls (IE86 and gelsolin, tracks 8 and 7 respectively).

At least in vitro, then, these data present a model by which IE86 could be recruited to the pre-RC complex through an interaction with Cdt1 which is then able to recruit MCM3AP to the origin of replication in an untimely fashion and, thereby, inhibit DNA synthesis.

To test this directly, we firstly analysed the ability of IE86 to interact with MCM3AP in vivo by analysing their co-localisation in infected cells (Figure 3B) as well as by immunoprecipitation (Figure 3C). Figure 3B shows that, following virus infection of GFP-tagged MCM3AP transfected cells, punctate MCM3AP staining is observed in the nucleus of HCMV infected cells which co-localises with IE86. Figure 3C also shows that in fibroblast cells expressing IE86 and GFP-tagged MCM3AP, immunoprecipitation of MCM3AP results in co-precipitation of IE86.

On the basis that MCM3AP was initially suggested to be an MCM3 acetylation factor, we tested whether HCMV infection or over-expression of IE86 could lead to increased acetylation of MCM3. Despite extensive analysis, we have been unable to show any robust increase in acetylation of endogenous MCM3 in response to arrest of cellular DNA synthesis by IE86. Consequently, the exact mechanism by which this recruitment of MCM3AP by IE86 inhibits DNA synthesis is, at present, unclear. However, it should be noted that strong acetyltransferase activity of endogenous MCM3AP has always been difficult to show and the evidence for this activity has previously relied on over-expression studies. It remains possible that acetylation is not its main mode of action.

### Viral IE86 causes the relocalisation of MCM3AP to the nucleus

Takei *et al.*, 2002 showed that over-expressed MCM3AP displays cytoplasmic punctate staining in cells. However, in the presence of MCM3, MCM3AP is re-localised to the nucleus via a direct interaction with MCM3 protein. Therefore, we asked whether IE86 could also cause a change in the intracellular localization of MCM3AP from the cytoplasm to the nucleus.

**Figure 4:**
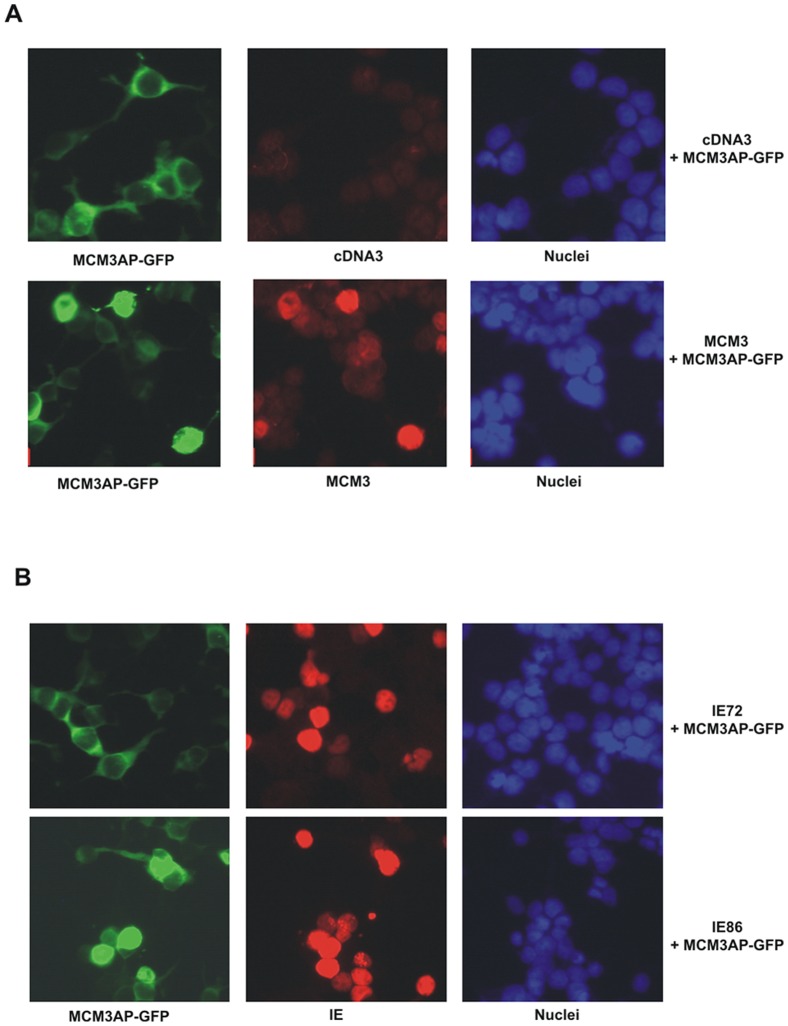
Viral IE86, like cellular MCM3, causes relocalisation of MCM3AP to the nucleus. 293-T cells were co-transfected with GFP-tagged MCM3AP (A and B) together with MCM3 or pCDNA3 (A); pCDNA3-IE72 (IE72) or pCDNA3-IE86 (IE86) (B). After fixing, MCM3AP-GFP expressing cells were analysed by co-staining for expression of MCM3, IE86 (IE) or IE72 (IE) as indicated. DNA (Hoechst 33342) staining is also shown.

Figure 4A shows that cells expressing GFP-MCM3AP alone displayed punctate cytoplasmic expression of MCM3AP. Consistent with previous observations, simultaneous over-expression of MCM3 resulted in relocalisation of MCM3AP into the nucleus. Interestingly, over-expression of IE86 also resulted in this same nuclear relocalisation of MCM3AP (Figure 4B, bottom panel). No such nuclear relocalisation of MCM3AP was observed after co-expression with IE72 (Figure 4B, top panel) or a mutant MCM3AP which is not relocalised to the nucleus by MCM3 [34] (data not shown). These data are consistent with the view that that IE86 causes an untimely relocalisation of MCM3AP to the nucleus where it mediates inhibition of cellular DNA synthesis.

### Inhibition of cellular MCM3AP expression abrogates the ability of viral IE86 to cause cell cycle arrest

To test directly whether MCM3AP is important for the inhibition of cellular DNA synthesis by IE86 we analysed the effect of IE86 on cell cycle in cells depleted of MCM3AP by RNAi technology.

**Figure 5:**
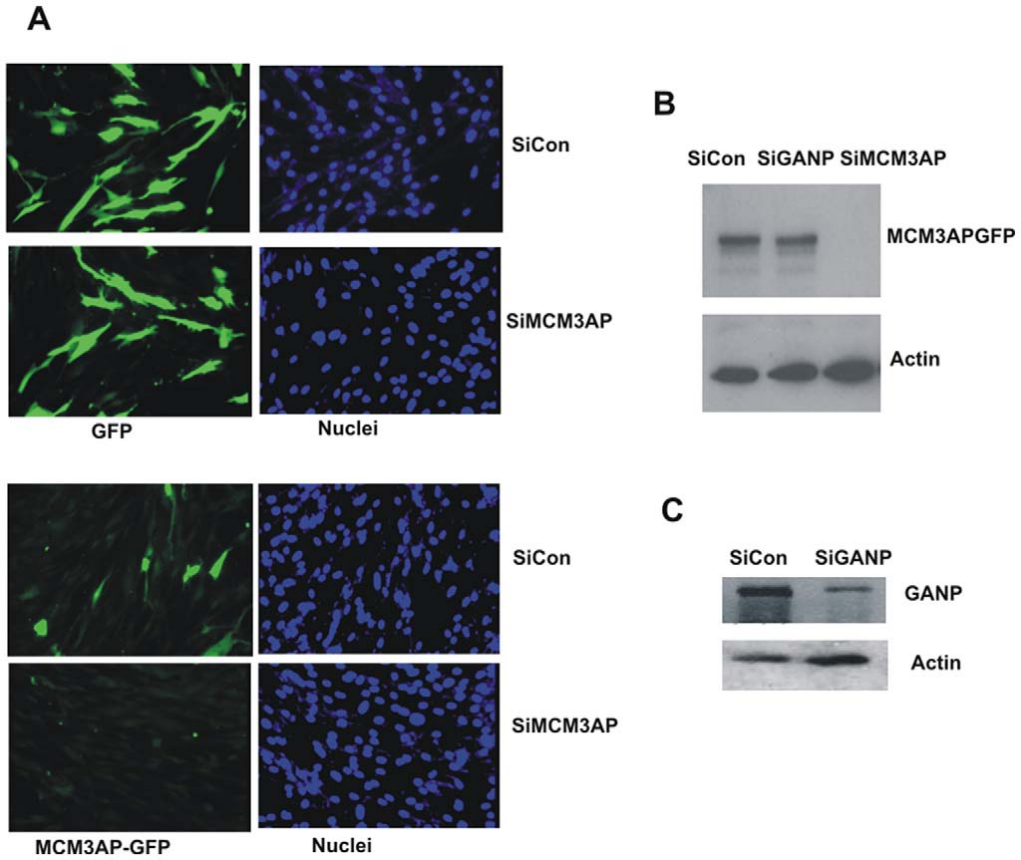
Knock down of MCM3AP protein by specific siRNAs. (A) HFFF cells were co-transfected with pCDNA3-GFP (GFP) or GFP-tagged MCM3AP (MCM3AP-GFP) together with an siRNA to MCM3AP (siMCM3AP) or a control siRNA (siCon) as indicated for 24 h. Transfected cells were analysed for GFP expression by immunofluorescence for GFP together with DNA counterstaining (Hoechst 33342) (B) HFFF cells transfected with GFP-tagged MCM3AP together with control siRNA (siCon), GANP siRNA (siGANP) or MCM3AP siRNAs (siMCM3AP) and analysed by western blotting with anti-GFP antibody to detect GFP-tagged MCM3AP expression. Actin loading controls are also shown (B). (C) Alternatively, cells were treated with siRNA (siCon) or GANP specific siRNA (siGANP) for 24 h, then analysed by western blot for GANP protein levels and actin (C).

HFFF cells treated with anti-MCM3AP siRNAs showed extremely good knock-down of expression of transfected GFP-tagged MCM3AP (Figure 5A bottom panel) whereas expression of a GFP control vector was unaffected (Figure 5A top panel). We also tested this effect of siRNAs on the expression of GFP-tagged MCM3AP in fibroblasts by western blot analysis which confirmed the ability of these siRNAs to target MCM3AP in contrast to a GANP-specific siRNA (Figure 5B).

**Figure 6:**
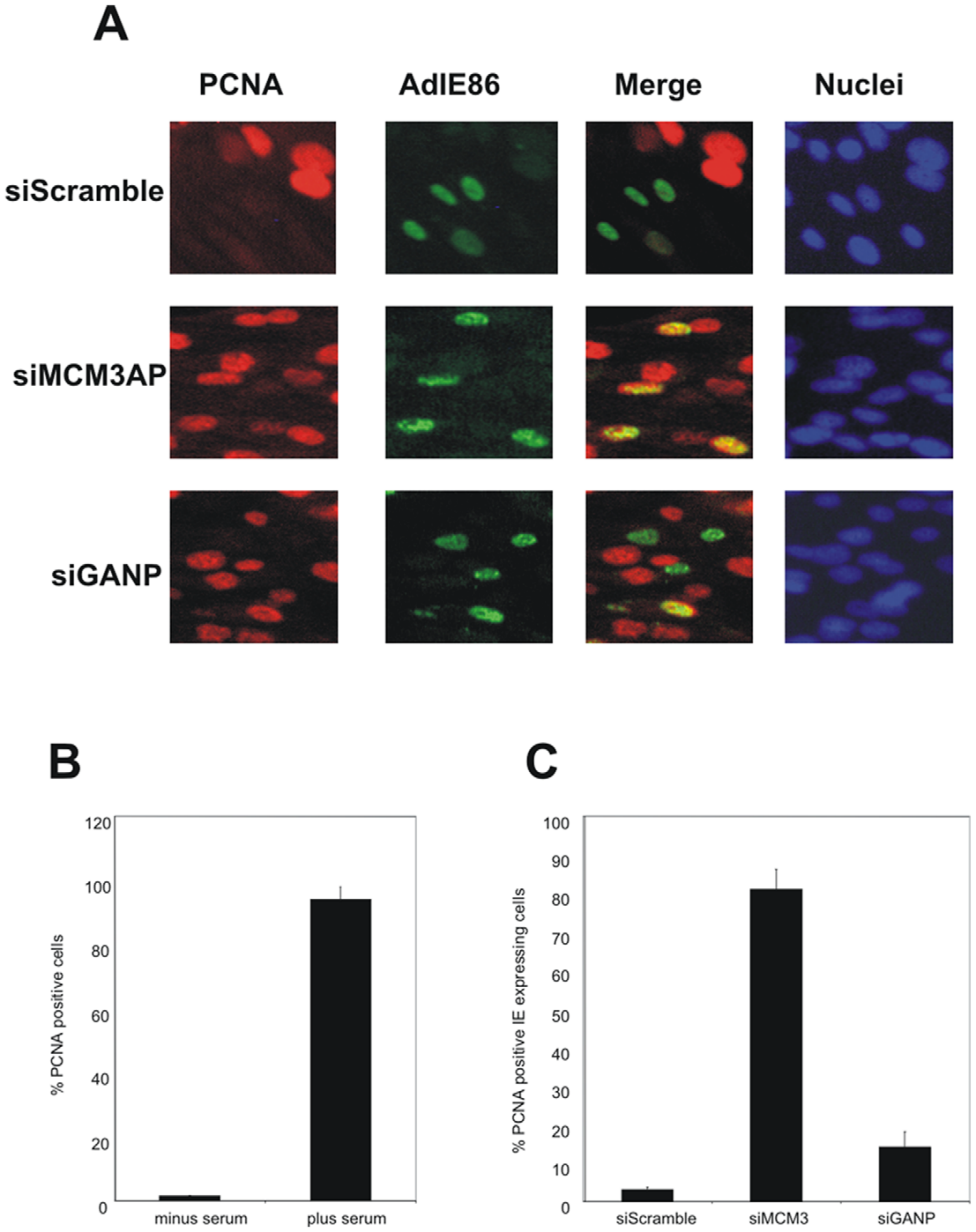
The inhibition of cellular MCM3AP expression prevents cell cycle arrest by viral IE86. (A) HFFF cells were transfected with siRNAs to MCM3AP (siMCM3AP) or GANP (siGANP) or a control siRNA (siCon) for 24 h in the absence of serum and subsequently infected with AdIE86 (approximately 1 pfu/cell) in the presence of serum for 24 h as indicated. Cells were then fixed and co-stained for IE protein (green) and PCNA (red). (B) Serum starved HFFF cells (−S) were treated with serum (+S) and the percentage of PCNA positive cells enumerated after (Hoechst 33342) counterstaining. (C) HFFFs were infected with AdIE86 (1 pfu/cell) in the presence of serum and transfected with siRNAs to MCM3AP or GANP (siMCM3AP, siGANP) or with control siRNA (siCon) as indicated. Cells were then stained with anti-PCNA antibody and nuclei were counterstained with (Hoechst 33342) The percentage of IE expressing cells that were PCNA positive were quantified (results shown are averages with standard deviations from the mean of 5 independent fields of view).

Consequently, we next tested the effect on MCM3AP knock-down on the ability of IE86 to inhibit cellular DNA synthesis. As we also wanted eventually to test this effect in the context of virus infection (see below), we used detection of insoluble PCNA, which acts as a robust direct surrogate for BrdU incorporation [35]–[38] as a marker of cellular DNA synthesis because BrdU incorporation cannot be used to assay cellular DNA synthesis in the context of HCMV infection for two reasons. First, viral DNA synthesis generates a high background, obscuring cellular synthesis and second, HCMV is known to prevent cellular thymidine salvage and to channel exogenous thymidine exclusively into viral DNA [39]. Consistent with this, Figure 6B shows that serum induction results in increased cellular DNA synthesis as detected by presence of insoluble PCNA. Figure 6A (and data quantified in Figure 6C) shows that cells treated with control siRNAs and expressing IE86 by adenoviral transduction were still inhibited for cellular DNA synthesis in that few, if any, IE86 expressing cells showed PCNA staining. In contrast, expression of IE86 in cells which had been treated with siRNAs targeting MCM3AP now resulted in IE86 expressing cells which were clearly undergoing DNA synthesis (Figure 6A and quantified in Figure 6C).

However, the gene encoding MCM3AP is known to be encoded entirely within a much larger sequence encoding the cellular protein GANP (germinal center-associated nuclear protein) gene (Wickramasinghe et al 2010a). Consequently, any siRNAs to MCM3AP will also target GANP protein expression [40]. Therefore, to ensure that the reversal of IE86-mediated inhibition of cellular DNA synthesis was specifically due to MCM3AP reduction and not a simultaneous knock-down of GANP, we also tested siRNAs which would target GANP but not MCM3AP. Figure 5C confirms that siRNAs specific to GANP did result in good knock-down of cellular GANP by western blot analysis but importantly that these GANP specific siRNAs had no effect on expression of MCM3AP (Figure 5B). Figure 6A (quantified in Figure 6C) shows that, in cells treated with siRNAs which target GANP but not MCM3AP, IE86 expression still profoundly decreases cellular DNA synthesis. Consequently, it appears that functions of MCM3AP, specifically, are required for IE86-mediated inhibition of cellular DNA synthesis.

**Figure 7:**
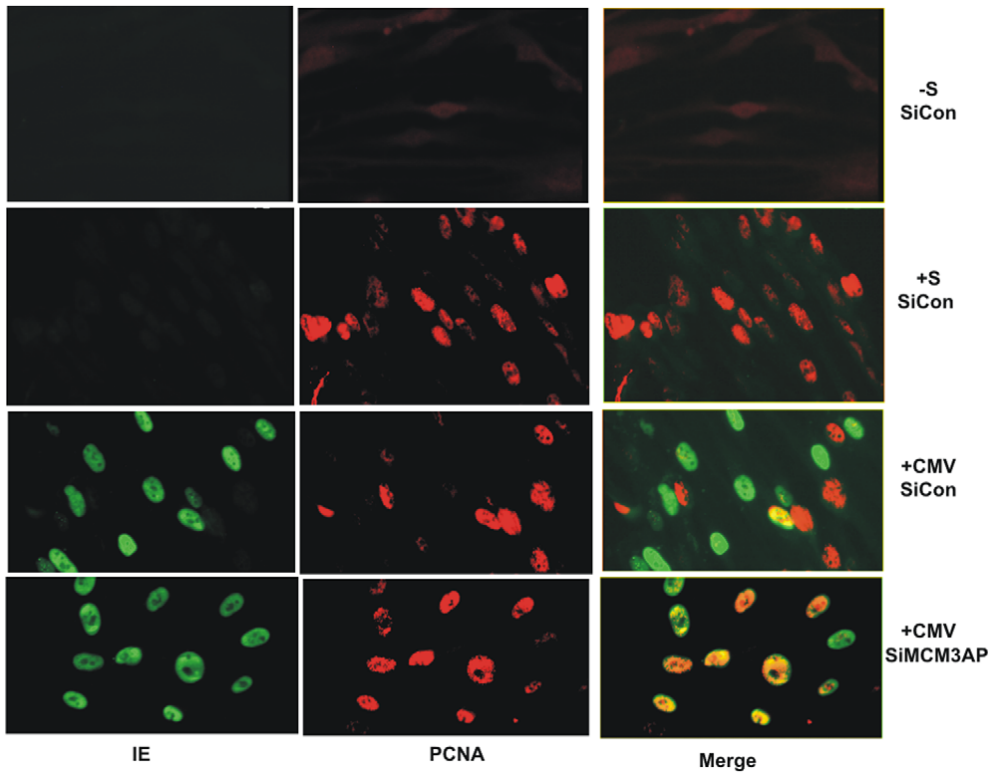
The inhibition of cellular MCM3AP expression prevents cell cycle arrest by HCMV infection. Cells were transfected with control siRNA (siCon) or MCM3AP siRNAs (siMCM3AP) for 24 h in the absence of serum. These cells were then infected overnight with HCMV (CMV) in the presence of serum. Additionally, siCon treated cells were either left overnight in serum free medium (−S) or treated with serum overnight (+S). Cells were then stained for IE expression (green) and PCNA (red).

Finally, we confirmed that knock-down of MCM3AP could also affect the ability of virus to inhibit cellular DNA synthesis in the context of virus infection. Figure 7 shows that, as before, treatment of cells with siRNAs to MCM3AP resulted in an inability of HCMV to inhibit cellular DNA synthesis at IE times of infection. No such effect was observed for cells treated with control siRNAs.

Taken together these results support the view that the ability of IE86 to inhibit cellular DNA synthesis at IE times of infection is mediated by cellular MCM3AP and it occurs at a step later than licensing of cellular origins of replication.

## Discussion

The targeting of cell cycle by HCMV is crucial for efficient lytic infection and HCMV encodes multiple factors which positively and negatively regulate host DNA replication: this likely ensures an optimum environment for viral DNA replication [6], [12], [18], [21], [23], [25]. IE72, IE86, pp71, and pUL97, when individually over-expressed, are known to inactivate pRb-family proteins resulting in the activation of E2F-dependent S-phase gene expression, which promotes a G1/S-transition. As with other DNA viruses, this strategy enables the host cell to support high levels of DNA replication. On the other hand, expression of pUL69 [41] or IE86 [4] is able to block cellular DNA synthesis. Although the mechanism by which pUL69 inhibits cellular DNA synthesis is unclear. It is likely that the subsequent arrest of cell cycle after promotion of the G1/S-transition ensures that the cell environment, once optimised for DNA replication, is forced to support viral and not cellular DNA replication. [12], [11], [39], [6], [18].

The IE86 protein of HCMV, then, appears to play a dual role in cycle regulation. It appears to induce the ATM-p53-p21 checkpoint [6] and can bind directly to p21 [8], Additionally, IE86 causes the upregulation of Cyclin E and other E2F target genes at the mRNA level [42] as well as activating the Cyclin E promoter [10]. It initially promotes a G_1_/S phase transition of resting cells but then mediates arrest of cell cycle and cellular DNA synthesis in early S-phase and the ability of IE86 to inhibit cellular DNA synthesis is essential for efficient viral DNA replication [12]. Our data now show that this effect of IE86 on cellular DNA synthesis at immediate early times of infection does not involve inhibition of cellular origin licensing but appears to occur at a post-licensing step.

In cells expressing IE86, which were clearly arrested in cellular DNA synthesis, we observed a physical interaction between IE86 and the cellular licensing factor Cdt1 in vitro and in vivo. However, we could observe no inhibition of licensing in these arrested cells: Cdt1 and MCMs were still efficiently loaded onto cellular chromatin.

We did, however, also observe an interaction between IE86 and the MCM3 associated protein MCM3AP and the ability of IE86 to recruit MCM3AP from the cytoplasm into the nucleus.

It is now clear that, whilst licensing of replication origins is an important step in the regulation of cellular DNA synthesis, this regulation can also occur at post-licensing steps. For instance, post-translational modification of MCMs by phosphorylation is needed to activate pre-RCs [43]–[47]. A role for the MCM3 associated protein MCM3AP in inhibition of initiation of cellular DNA synthesis in a licensing-independent manner has also been described (Takei et al 2002).

Consistent with an important role for MCM3AP in IE86-mediated suppression of cellular DNA synthesis, IE86-expressing cells in which MCM3AP expression had been inhibited by siRNAs no longer showed this inhibition. Importantly, cells infected with HCMV after MCM3AP knock-down were also still able to synthesise cellular DNA.

We think it likely that during the course of HCMV infection multiple viral functions sequentially target cellular DNA synthesis. Our observations suggest that, at immediate early times of infection, IE86 prevents MCM function by recruitment of MCM3AP which prevents the pre-RC from initiating DNA synthesis. However, at later times of infection, other viral mechanisms take over this responsibility to ensure the cell is constantly held in pseudo-G_1_ phase of cell cycle. It is interesting to note that Qian et al [23] have recently shown that the pUL117 early viral protein is able to inhibit MCM loading onto chromatin at early times of infection also resulting in the inhibition of cellular DNA synthesis.

In conclusion, we have identified the cellular factor MCM3AP as being essential for inhibition of cellular DNA synthesis by IE86 at IE times of HCMV infection in primary fibroblasts. Our observations suggest that IE86-mediated recruitment of MCM3AP to the nucleus likely results in the untimely delivery of inhibitory MCM3AP to replication complexes and provides a novel mechanism by which HCMV inhibits cellular DNA synthesis.

## Materials and Methods

### Cells and viruses

HEK293T cells and human foetal foreskin fibroblasts (HFFF) cells were obtained from the European Collection of Cell Cultures (ECACC) and maintained in minimal essential medium containing 10% foetal calf serum (MEM-10) as described previously [48]. Cells were infected with human cytomegalovirus (AD169 strain) at an m.o.i. of 5. Alternatively, cells were infected with a recombinant HCMV expressing an IE86-EYFP fusion [49]. Adenovirus vectors expressing either the tetracycline-inducible “Tet-off” transactivator (AdTrans), the viral IE2-p86 protein (AdIE86), or green fluorescent protein (AdGFP) and their use have been described previously [4]. Routinely, cells were transduced with an equal amount (1–5 pfu/cell) of each of AdTrans with AdGFP or an equal amount (1–5 pfu/cell) of each of AdTrans with AdIE86 for 36–48 h. Prior to infection with HCMV or transduction with adenovirus, cells were synchronised by serum starvation for 24 h. Ad Cdt1 has been described previously [50].

### Plasmids and cell transfection

MCM3, GFP-tagged MCM3AP, untagged MCM3AP and GFP-tagged MCM3AP mutant expression vectors have all been described previously [30], as have empty pCDNA3 and pCDNA3-based expression vectors for HCMV IE72 and IE86 [51].

To analyse relocalisation of MCM3AP by MCM3 or IE86, HEK293T cells were transfected with GFP-tagged MCM3AP or GFP-tagged MCM3AP mutant expression plasmids together with MCM3 or IE86 expression vectors using Lipofectamine 2000 (Invitrogen) as described by the manufacturer. Cells were fixed in 4% paraformaldehyde and permeabilised with 0.1% Triton-X-100 to maintain GFP fluorescence and then co-stained with mouse monoclonal antibodies to MCM3 (Stressgen) or IE antibody (E13, Biosys) which were detected with donkey anti-mouse Alexafluor 488/592.

To analyse co-localisation of IE86 and MCM3AP-GFP in infected cells, a mouse anti-IE86 specific antibody which works well in immunoflurescence analyses, but weakly in western blot analyses [52] was used which was detected by rabbit anti-mouse TRITC.

### Protein expression and GST-fusion pulldowns

pGEX-3X.IE2, used to generate recombinant IE86 protein, has been described previously [51]. Recombinant GST or GST-fusion proteins were purified by using glutathione sepharose beads and eluted with glutathione [53].

Protein-protein interaction assays were carried out exactly as described previously [51]. Vectors to generate [^35^S]-methionine-labelled IE72, IE86, and gelsolin by coupled *in vitro* transcription}translation (Promega) have also been described previously [51]

### Immunofluorescence for DNA replication analyses

For 5-bromo-2′-deoxyuridine (BrdU) incorporation assays, cells were treated for 24 h with 10 uM BrdU at 24 h post-infection (p.i.) as described previously [54] Briefly, cells were fixed in 4% paraformaldehyde for 15 min and nucleic acids were denatured with 2 N HCl for 1 h. After neutralisation for 20 min in 0.1 M Na_2_B_4_O_7_ (pH 8.5), cells were then permeabilized in 70% ethanol at −20°C and then co-stained with mouse monoclonal antibodies to IE (Biosys), rat monoclonal antibodies to BrdU (Serotec) or a rabbit polyclonal antibody to GFP (Abcam). Antibodies were detected using a goat anti-mouse FITC conjugate for IE, a sheep anti-rabbit FITC conjugate for GFP and a rabbit anti-rat TRITC conjugate for BrdU. To counterstain nuclei, slides were incubated for 5 min in 0.1 µg/ml of DAPI (4′,6′-diamidino-2-phenylindole). Alternatively, to assess cellular DNA replication, cells were fixed with acetone/methanol as previously described [35] which specifically detects replication-associated PCNA. After PCNA staining with a mouse anti-PCNA antibody (Santa Cruz), cells were stained with an anti-IE antibody as described above. To counterstain nuclei, slides were incubated for 5 min in 1 µg/ml of Hoechst 33342.

### siRNA knockdown analyses and RT-PCR

For the knockdown of MCM3AP, two siRNA molecules were designed:

5′-GGCGGCUCAGAAACAAGAC – 3′ and 5′-GUUCAUGGGAGAUGAAGGC-3′ and for GANP specific siRNA molecules 5′ AAAUAGUGAACUCUGGUUUUGG-3′ and 5′-GCAAGAACAUCCCUGACUACC-3′ (Dhamacon) or the scramble control siRNA (Dharmacon) and siRNA transfections were performed using a combination of both siRNAs with Lipofectamine 2000 (Invitrogen) as described previously [55]. RT-PCRs were carried out as described previously [56] using the Promega RT kit following the manufacturer instructions followed by PCR using the PCR primers 5′-CCGCGCCTGACTGACTCTTG-3′ and 5′-CTGAGCCTTCATCTCCCATG-3′ or as described previously [57].

### Immunoprecipitation and Western blot analyses

To assay recruitment of cell cycle-associated proteins to cellular chromatin, chromatin-associated proteins were isolated as previously described [30] and analysed by SDS-PAGE on 10% acrylamide gels. After blotting, proteins were detected with antibodies to MCM7 (Mouse monoclonal, Sigma), MCM3 (Rabbit polyclonal, Abcam), Cdt1 (rabbit polyclonal; a kind gift of Dr Wu, Scripps Institute), HCMV IE (E13, Biosys), adenovirus type 5 hexon protein (Rabbit polyclonal, Abcam) followed by anti-mouse or anti-rabbit HRP conjugated secondary antibody as appropriate for western blot detection.

For immunoprecipitations followed by western blot (IP-western), cells were infected with AdCdt1 with or without co-infection with HCMV or recombinant HCMV expressing EYFP-tagged IE86 as described above and immunoprecipitated as described previously [58] with anti-Cdt1 antibody (rabbit polyclonal; Abcam 22716) or a rabbit control antibody which was followed by western blot analysis of co-precipitated IE proteins with E13 antibody (Biosys). The same samples were also probed by western blot with an IE1-specific antibody (Vancouver Biotech Ltd; 6E1).

To analyse knock-down of endogenous GANP, a GANP-specific antibody was used as described previously [59].
